# Optimizing cardiac diffusion tensor imaging in vivo: More directions or repetitions?

**DOI:** 10.1016/j.jocmr.2025.101951

**Published:** 2025-09-02

**Authors:** Sam Coveney, David Shelley, Richard J. Foster, Maryam Afzali, Ana-Maria Poenar, Noor Sharrack, Sven Plein, Erica Dall’Armellina, Jürgen E. Schneider, Christopher Nguyen, Irvin Teh

**Affiliations:** a Leeds Institute of Cardiovascular and Metabolic Medicine (LICAMM), University of Leeds, Leeds, UK; b Leeds Teaching Hospitals NHS Trust, Leeds, UK; c Cardiff University Brain Research Imaging Centre (CUBRIC), School of Psychology, Cardiff University, Cardiff, UK; d Cleveland Clinic, Heart Vascular Thoracic Institute, Cardiovascular Innovation Research Center, Cleveland, Ohio, USA

**Keywords:** Cardiac MRI, Diffusion tensor imaging, Diffusion schemes, Diffusion encoding directions, Heart, Myocardium

## Abstract

**Background:**

Cardiac diffusion tensor imaging (cDTI) is sensitive to imaging parameters, including the number of unique diffusion encoding directions (ND) and number of repetitions (NR; analogous to number of signal averages). However, there is no clear guidance for optimizing these parameters in the clinical setting.

**Methods:**

Spin echo cDTI data with second-order motion-compensated diffusion encoding gradients were acquired in 10 healthy volunteers on a 3T magnetic resonance imaging scanner with different diffusion encoding schemes in pseudo-randomized order. The data were subsampled to yield 96 acquisition schemes with 6 ≤ ND ≤ 30 and 33 ≤ total number of acquisitions (NA_all_) ≤ 180. Stratified bootstrapping with robust fitting was performed to assess the accuracy and precision of each acquisition scheme. This was quantified across a mid-ventricular short-axis slice in terms of root mean squared difference (RMSD), with respect to the full reference dataset, and standard deviation (SD) across bootstrap samples, respectively.

**Results:**

For the same acquisition time, the ND = 30 schemes had on average 48%, 40%, 34%, and 34% lower RMSD and 6.2%, 7.4%, 10%, and 5.6% lower SD in mean diffusivity (MD), fractional anisotropy (FA), helix angle (HA), and absolute sheetlet angle (|E2A|) compared to the ND = 6 schemes. Given a fixed number of high b-value acquisitions, there was a trend toward lower RMSD and SD of MD and FA with increasing numbers of low b-value acquisitions. Higher NA_all_ with longer acquisition times led to improved accuracy in all metrics, whereby quadrupling NA_all_ from 40 to 160 volumes led to *a* 20%, 39%, 11%, and 5.4% reduction in RMSD of MD, FA, HA, and |E2A|, respectively, averaged across six diffusion encoding schemes. Precision was also improved with *a* corresponding 53%, 50%, 53%, and 36% reduction in SD.

**Conclusion:**

We observed that accuracy and precision were enhanced by (i) prioritizing number of diffusion encoding directions over NR given a fixed acquisition time, (ii) acquiring sufficient low b-value data, and (iii) using longer protocols where feasible. For clinically relevant protocols, our findings support the use of ND = 30 and NA_b50_:NA_b500_ ≥ 1/3 for better accuracy and precision in cDTI parameters. These findings are intended to help guide protocol optimization for harmonization of cDTI.

## Graphical abstract

**Figure:**
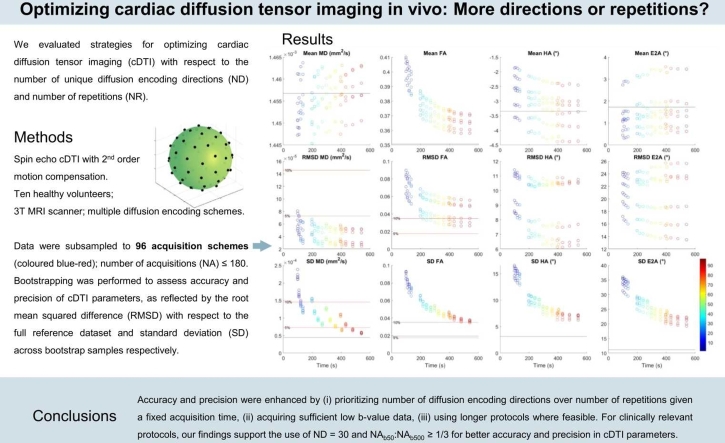

## 1 Introduction

Cardiac diffusion tensor imaging (cDTI) is a rapidly emerging technique for myocardial tissue characterization in vivo without the need for contrast agents [1]. It has shown promise in characterizing the microstructural changes in several clinical scenarios, including myocardial infarction [2], [3], [4], [5], [6], hypertrophic cardiomyopathy [7], [8], [9], [10], [11], aortic stenosis [12], amyloidosis [13], and others. The major challenge of cDTI for in vivo imaging is its high sensitivity to bulk motion of the heart during contracture. To ameliorate this, the use of second-order motion-compensated (M2) diffusion encoding gradients has become standard practice in spin echo planar imaging (EPI) [14], [15]. This leads to longer echo times (TEs) and lower signal-to-noise ratio (SNR) compared to using non-motion-compensated diffusion gradients, which is compounded by the relatively short T_2_ of the myocardium, i.e., ∼44 ms at 3T [16].

To obtain adequate SNR and to mitigate instances of images containing artifacts, clinical cDTI protocols typically employ high number of repetitions (8 ≤ NR ≤ 16) [14], [17], [18]. We refer to NR instead of the more commonly used number of signal averages because cDTI data are in general exported and reconstructed offline as separate repetitions instead of being averaged on the scanner. However, the number of unique diffusion encoding directions (6 ≤ ND ≤ 12) [14], [15], [17], [18], [19] often remains small relative to diffusion tensor imaging (DTI) protocols used in other anatomy, such as the brain, where ND ≥ 30 has been recommended [20]. As the imaging time is related to both NR and ND, identifying a suitable range of NR and ND is important for cDTI within a clinically feasible timeframe. Moreover, DTI parameters, including mean diffusivity (MD) and fractional anisotropy (FA), as measured in the healthy myocardium with spin echo methods are known to vary in the literature with reports of 0.75 × 10^−3^ mm^2^/s ≤ MD ≤ 1.72 × 10^−3^ mm ^2^/s and 0.29 ≤ FA ≤ 0.43 [17], [19], [21]. Known imaging sources of variation for these parameters include spatial resolution [22], ND, NR, SNR [23], diffusion encoding time [24], and b-value [18]. Isolating the effects of NR and ND is therefore an important aspect in the optimization of efficient and robust cDTI protocols for clinical use.

In DTI, ND = 6 is the minimum number of unique non-zero b-value acquisitions required for tensor estimation [25]. However, previous work in the brain investigating the dependence of DTI parameters on ND showed that higher ND reduced artifactually elevated FA [26], [27] and its standard deviation (SD) [26], particularly in regions of low FA [28]. Higher SNR, as may be obtained by averaging, led to similarly lower and more accurate FA [29], [30]. As a guide, ND ≥ 20 was recommended for measurement of FA [26], [31], [32] while ND ≥ 30 was suggested for measurement of tensor orientation and MD [26]. While the effects of ND and NR have been reported ex vivo [23], there have only been preliminary reports [33], [34], [35], [36] that provide no conclusive evidence on the requirements of ND and NR for cDTI in vivo. A recent consensus statement on cDTI published by the Society for Cardiovascular Magnetic Resonance Cardiac Diffusion Special Interest Group recommended that more than six directions should be used but highlighted that rationalizing ND and NR remain an unmet need in the development of cDTI [1]. Furthermore, there exist various approaches for sampling low b-value data in cDTI. While there are methods for optimizing the quantity of low b-value data as a proportion of high b-value data in the brain [37], optimal sampling of low b-value data is less well understood in the heart.

In this study, we investigated the accuracy and precision of several diffusion sampling schemes over a wide range of ND and NR in healthy volunteers, extending our previous ex vivo work [23]. We compare time-normalized data to assess the trade-offs made between ND and NR, and examine the effects of the number of low b-value acquisitions. We hypothesized that accuracy and precision in DTI measurements would be improved by (i) sampling schemes that prioritize ND over NR, (ii) sufficient sampling of low b-value data, and (iii) longer scan times. This is intended to inform optimization and standardization of clinical cDTI protocols.

## 2 Methods

### 2.1 Data acquisition

cDTI data were acquired in healthy volunteers (N = 10) using a Prisma 3T magnetic resonance imaging (Siemens Healthineers, Erlangen, Germany) with maximum gradient amplitude, Gmax = 80 mT/m and a combination of 18-channel body and 32-channel spine radiofrequency array coils. The study was performed under approved ethics, and healthy volunteers provided written informed consent. Data were acquired with single-shot spin echo EPI, two-dimensional radiofrequency inner volume excitation and cardiac triggering: repetition time (TR) = 3 RR-intervals, TE = 76 ms, in-plane resolution = 2.3 × 2.3 mm ^2^, slice thickness = 8 mm, number of slices = 3, field-of-view = 320 × 111 mm ^2^, partial Fourier = 7/8, bandwidth = 2012 Hz/px, b_low_ = 50 and b_high_ = 500 s/mm^2^. Up to second-order motion-compensated diffusion encoding gradient waveforms were applied. Subjects were scanned under free-breathing conditions without respiratory gating, in late systolic phase.

**Fig. 1:**
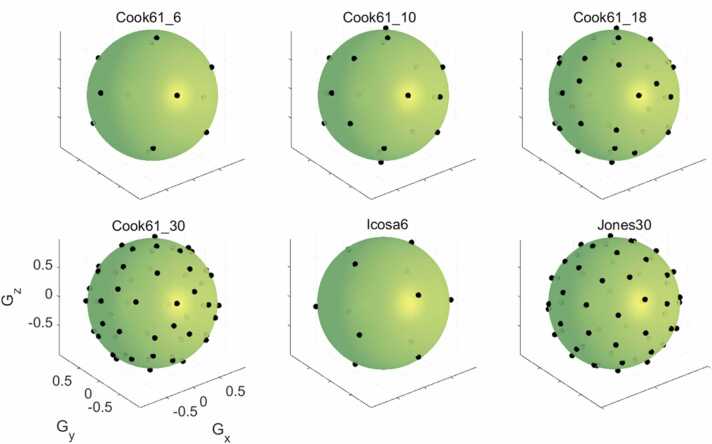
Diffusion encoding schemes investigated include Cook61 diffusion encoding scheme subsampled to 6, 10, 18, and 30 directions, a 6-direction icosahedral scheme and a 30-direction scheme by Jones et al. Each direction was reflected on the opposite side of the sphere

Diffusion-weighted data were acquired in pseudo-randomized fashion over a range of diffusion encoding schemes (DES) with different ND and NR. Low b-value data were acquired with ND = 3 orthogonal directions, denoted by ND_orth3,b50_. High b-value data were acquired with a 61-direction Cook DES [38] that was sequentially subsampled to 6, 10, 18, and 30 direction sets. These DES were denoted as Cook61_6, Cook61_10, Cook61_18, and Cook61_30. The Cook DES was particularly amenable to subsampling because it was optimized for incremental sampling in case of premature scan termination. Previous work has shown that noise performance (and therefore the accuracy of parameter estimates) of a given DES is significantly correlated with the condition number of its transformation matrix [39]. Specific sets of sequentially subsampled directions were identified to minimize the condition numbers of the transformation matrices. This was performed by circularly subsampling ND = 6, 10, 18, and 30 sequential directions from the Cook61 scheme, incrementing the first diffusion encoding direction (D_i_) from 1 to 61, and finding D_i_ that minimized the condition numbers. For the DES subsampled to 6, 10, 18, and 30 directions, condition numbers were relatively low at 1.74, 1.75, 1.70, and 1.58, respectively. For reference, the condition numbers of the widely used Jones30 and 6-direction dual gradient schemes are 1.59 and 2.00, respectively [39]. An icosahedral DES [40] with six diffusion encoding directions (Icosa6) and Jones DES [37] with 30 diffusion encoding directions (Jones30) were also acquired. An upper limit of 30 diffusion encoding directions was specified due to diminishing returns with higher ND [29]. These DES are illustrated in Fig. 1.

The full set of data acquired included NR(ND_orth3, b50_) = 24, NR(ND_Cook61_30, b500_) = 12, NR(ND_Cook61_6, b500_) = 8, NR(ND_icosa6, b500_) = 20, and NR(ND_Jones30, b500_) = 4. This yielded a total number of acquisitions (NA_all_) = 24 × 3 + 12 × 30 + 8 × 6 + 20 × 6 + 4 × 30 = 720 volumes, which constituted the reference dataset acquired in a single scan. The reference dataset was subsampled into the six DES listed in Fig. 1, with the total number of high b-value acquisitions (NA_b500_) set to 30, 60, 90, and 120 volumes each. For a 6-direction scheme, this translated to NR = 5, 10, 15, and 20 repetitions, respectively. To keep acquisition times consistent, schemes with larger ND had proportionally lower NR. In cases where NA was not divisible by ND, e.g., Cook61_18, the last repetition of data would form an incomplete shell. Further to the DES, we also investigated the sensitivity of DTI to low b-value acquisitions. Here, we sampled the number of low b-value acquisitions (NA_b50_) equal to a factor of the number of high b-value acquisition (NA_b500_), where NA_b50_ = NA_b500_/[10, 5, 3, 2]. For example, in a 6-direction scheme with NA_b500_ = 30, NA_b50_ = 3, 6, 10, and 15. The total number of acquisitions (NA_all_) was the sum of NA_b50_ and NA_b500_. This yielded 96 subsampled combinations, i.e., acquisition schemes comprising 6 DES, 4 sets of low b-value acquisitions, and 4 sets of high b-value acquisitions, as reflected in Table 1. The naming convention follows the format “DES_NA_b500__NA_b50_,” e.g., Cook61_30_90_30. Nominal acquisition time = NA_all_ × nominal TR = 720 × 3 s = 36 min, where nominal TR = number of slices × 60/heart rate, where data were acquired in a slice-interleaved manner and with assumed heart rate of 60 bpm.

**Table 1:** Acquisition schemes extracted from the reference dataset, indicating numbers of diffusion encoding directions for high b-value data (ND_b500_), numbers of acquisition volumes for high b-value data (NA_b500_), number of repetitions for high b-value data (NR_b500_), numbers of acquisition volumes for low b-value data (NA_b50_), including three orthogonal diffusion encoding directions, total numbers of acquisition volumes (NA_all_), and acquisition time.

Diffusion encoding scheme		Cook61_6/Icosa6				Cook61_10				Cook61_18				Cook61_30/Jones30			
ND_b500_		6	6	6	6	10	10	10	10	18	18	18	18	30	30	30	30
NA_b500_		30	60	90	120	30	60	90	120	30	60	90	120	30	60	90	120
NR_b500_	= NA_b500_/ND_b500_	5	10	15	20	3	6	9	12	1.7	3.3	5	6.7	1	2	3	4
NA_b50_	= NA_b500_/[10, 5, 3, 2]	3	6	9	12	3	6	9	12	3	6	9	12	3	6	9	12
		6	12	18	24	6	12	18	24	6	12	18	24	6	12	18	24
		10	20	30	40	10	20	30	40	10	20	30	40	10	20	30	40
		15	30	45	60	15	30	45	60	15	30	45	60	15	30	45	60
NA_all_	= NA_b50_ + NA_b500_	33	66	99	132	33	66	99	132	33	66	99	132	**33**	**66**	**99**	**132**
		36	72	108	144	36	72	108	144	36	72	108	144	**36**	**72**	**108**	**144**
		*40*	*80*	*120*	*160*	*40*	*80*	*120*	*160*	*40*	*80*	*120*	*160*	***40***	***80***	***120***	***160***
		45	90	135	180	45	90	135	180	45	90	135	180	**45**	**90**	**135**	**180**
Time (s)	= N_all_ * 3RR intervals (= 3 s @ 60 bpm)	99	198	297	396	99	198	297	396	99	198	297	396	99	198	297	396
		108	216	324	432	108	216	324	432	108	216	324	432	108	216	324	432
		120	240	360	480	120	240	360	480	120	240	360	480	120	240	360	480
		135	270	405	540	135	270	405	540	135	270	405	540	135	270	405	540

For brevity and clarity, subsets of data with different diffusion encoding schemes (highlighted italics) and NAb50 (highlighted bold) are reported in detail in Section 3

*NA* number of acquisitions, *ND* number of diffusion encoding directions, *NR* number of repetitions

### 2.2 Data analysis

Magnitude-only data were exported from the scanner and analyzed. Post-processing used in-house tools developed in Python. Image registration was performed by masking a suitable low b-value image using a square, registering all b_low_ images to this reference image, then using the average of registered b_low_ images as a reference image to register all images. The DTI signal representation was then fitted to the full dataset using robust weighted-least squares [41] implemented in DiPy [42], and the entire image series was predicted from this fit. All original images were then re-registered to these predicted images, leading to superior registration compared to the first stage. Rigid registration was performed using SimpleITK [43] with Mutual Information as a metric, calculated within the square mask. To assess the potential of signal rectification in the high b-value data, SNR_b500_ was calculated in a mid short-axis slice by taking the mean signal intensity over repetitions divided by the SD over repetitions [44] for each diffusion encoding direction in the registered data, and then averaging across directions and voxels.

Diffusion tensor fitting was performed on the registered images using robust weighted-least squares [41]. The robust fitting method has been previously shown to be superior to whole-image shot-rejection [41] and the weighted-least squares method mitigates signal rectification bias in magnitude images [45]. cDTI parameters MD, FA, helix angle (HA), and sheetlet angle (E2A) were calculated. HA and E2A were measured using a cylindrical coordinate system with origin at the center of mass of the left ventricular (LV) segmentation on a slice-wise basis, as defined here [46]. Segmentation of the LV contours was performed with care taken to exclude voxels exhibiting partial-volume effects. Regions affected by strong artifacts that may negatively impact the results were masked out by defining “sectors” centered on the LV blood pool such that these voxels are ignored in the voxel statistics.

To assess the relative performance of each acquisition scheme, we use bootstrapping to approximate the sampling distribution of diffusion measures, by using the full reference dataset to generate samples of possible datasets that could be obtained from each acquisition scheme. Bootstrapping was done using the repetition bootknife method [47] which is a form of stratified bootstrapping. Each diffusion encoding direction was treated as a stratum, and each bootstrap sample was generated by randomly choosing (with replacement) images from each stratum, after first removing a random image from each stratum. A total of 500 bootstrap samples were generated per acquisition scheme (Table 1). This was well in excess of the minimum number of bootstrap samples required for stable measurements of accuracy and precision, and in a similar range as in the previous literature [48], [49]. The number of images chosen from each stratum was based on the NR specified for each shell. Where NA_b500_ was perfectly divisible by ND_b500_, i.e., in most cases, these images were distributed equally across diffusion encoding directions for the current shell. Where NA_b500_ was not perfectly divisible by ND_b500_ (e.g., in the Cook61_18 scheme with NA_b500_ = 30), any remaining images were then assigned to random unique directions within the design for the current shell, so there was at most a difference of 1 image per stratum in the current shell.

For each individual DES, accuracy was assessed by calculating the root mean squared difference (RMSD) between the bootstrap samples and the full reference dataset. Precision was assessed by the SD of cDTI metrics across bootstrap samples, whereby a lower SD reflected higher precision, i.e., precision = (SD)^−2^. The mean, RMSD, and SD were then averaged over voxels in segmented regions-of-interest in a mid-myocardial slice and in the mid-ventricular septal wall, i.e., American Heart Association regions 8 and 9, to exclude regions with poorer B_0_ homogeneity, e.g., near the posterior vein. Boxplots of mean, RMSD, and SD are presented with median and interquartile range (IQR) over subjects. Individual bootstrap sample data in all volunteers from selected DES were also presented as histograms. The data were fitted using normal distributions. Non-overlapping 95% confidence intervals (CI) of the mean indicate statistically significant differences between acquisition schemes. Paired t-tests for RMSD and SD measures were performed between each acquisition scheme. P-values were adjusted for multiple comparisons using Bonferroni-Holm correction [50] with a significance threshold of p < 0.05. P-value matrices were calculated, and cDTI parameters compared against nominal imaging time.

## 3 Results

Subject characteristics were age = 23 ± 4 years, 1 male, 9 females, average heart rate = 71 ± 10 beats per minute (bpm), weight = 61 ± 10 kg, and body mass index = 22 ± 2 (mean ± SD across subjects). SNR_b500_ was 9.6 ± 1.0 (mean ± SD across subjects). An example of image quality and registration performance is given (Supplementary Video 1).

Supplementary material related to this article can be found online at doi:10.1016/j.jocmr.2025.101951.

The following is the Supplementary material related to this article Video S2..

Video S1 Supplementary material related to this article can be found online

**Fig. 2:**
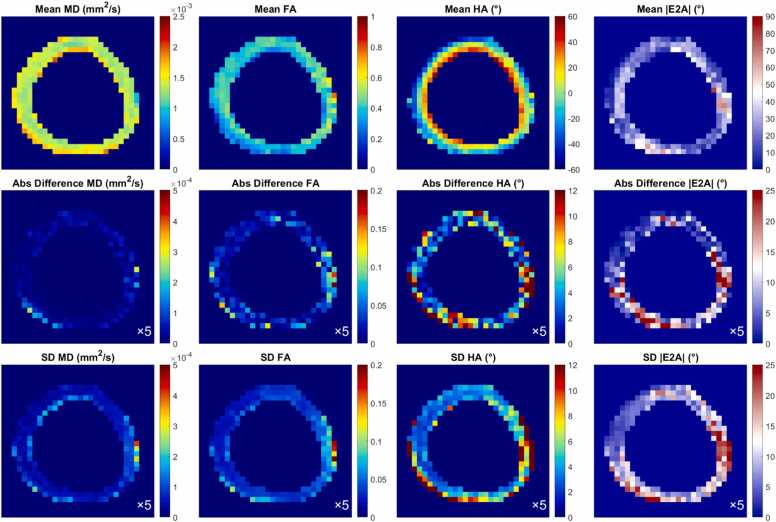
cDTI maps MD, FA, HA, and |E2A| in *a* mid-myocardial short-axis slice in *a* representative healthy volunteer using the example diffusion encoding scheme Cook61_30 with NA_b500_ = 90 and NA_b500_ = 30. (Top) Mean over bootstrap samples, (middle) absolute difference between current and reference diffusion encoding scheme, and (bottom) standard deviation across bootstrap samples. Region of elevated absolute difference and SD in the inferolateral wall is indicated by arrows. These maps were scaled at 5× smaller range to highlight the heterogeneity. *cDTI* cardiac diffusion tensor imaging, *MD* mean diffusivity, *FA* fractional anisotropy, *HA* helix angle, *|E2A|* absolute sheetlet angle, *NA* number of acquisitions, *SD* standard deviation

MD, FA, HA, and |E2A| maps in a representative volunteer are shown (Fig. 2). The maps are consistent with those reported in the literature. Absolute differences with respect to the reference data and SD were elevated in the inferolateral wall, corresponding to the region near the posterior vein, but this effect was less distinct in the average parameter maps.

**Fig. 3:**
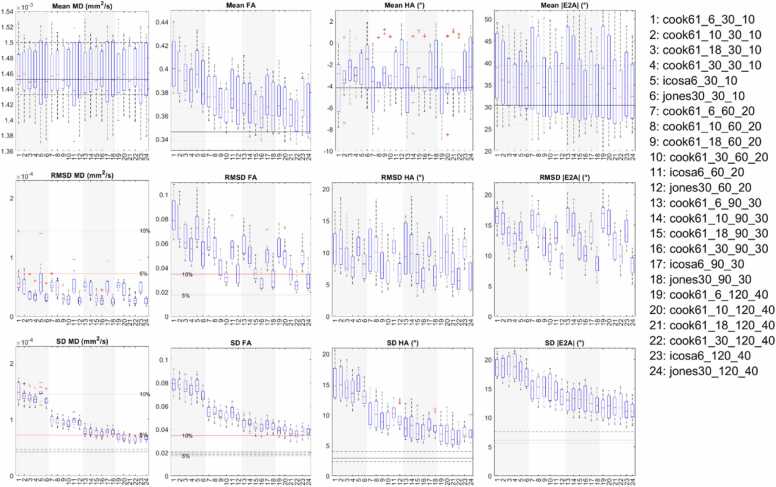
Boxplots of cDTI metrics (left to right) MD, FA, HA, and |E2A| showing (top) cDTI metrics averaged over bootstrap samples, (middle) RMSD with respect to the fully sampled reference data, and (bottom) SD across bootstrap samples across a mid-myocardial short-axis slice. Twenty-four acquisition schemes are described in the following format “DES_NA_b500__NA_b50_,” e.g., Cook61_30_90_30. These were sorted by diffusion encoding scheme and grouped into four groups (white and gray vertical bands) with increasing NA_all_ corresponding to increasing acquisition times. For reference, median and IQR values from the reference dataset are given (black solid and dashed lines); 5% and 10% of the median MD and FA from the reference dataset are indicated (red solid lines). *cDTI* cardiac diffusion tensor imaging, *MD* mean diffusivity, *FA* fractional anisotropy, *HA* helix angle, *|E2A|* absolute sheetlet angle, *RMSD* root mean squared difference, *NA* number of acquisitions, *SD* standard deviation, *DES* diffusion encoding schemes, *IQR* interquartile range

Boxplots reflecting (i) cDTI metrics averaged over bootstrap samples, (ii) accuracy of cDTI metrics expressed as the RMSD with respect to the fully sampled reference data, and (iii) 1/sqrt(precision) as expressed by SD across bootstrap samples across a mid-myocardial short-axis slice are shown (Fig. 3; median and IQR over subjects). For clarity of presentation, we focused first on 24 acquisition schemes with 6 different DES and 4 different total acquisition times. In all cases, NA_b500_:NA_b50_ = 3. For context, these schemes are highlighted in yellow in Table 1. In the reference data, mean MD = (1.46 ± 0.04) × 10^−3^ mm^2^/s, mean FA = 0.35 ± 0.02, mean HA = −3.4° ± 3.0°, mean |E2A| = 32.9° ± 10.0° (mean ± SD across subjects). Within each band with normalized acquisition times in Table 1, there was a small but observable trend toward lower RMSD and SD in MD, FA, HA, and |E2A| with higher ND, indicating higher accuracy and precision. This was consistent across Cook, Icosa, and Jones DES. For the same acquisition time, for the ND = 30 schemes (Cook and Jones), RMSD MD, FA, HA, and |E2A| were on average 48%, 40%, 34%, and 34% lower than for the ND = 6 schemes (Cook and icosahedral). Similarly, SD of MD, FA, HA, and |E2A| were 6.8%, 7.8%, 10%, and 1% lower in the former compared to the latter. Mean FA was most sensitive to DES, while MD, HA, and |E2A| were less so.

Across bands with different acquisition times, there was a clear trend toward lower SD (i.e., better precision) in all cDTI metrics with increasing number of acquisitions and scan time. For ease of reference, these results were reformatted into bands with different DES and ordered by increasing NA_all_ within each band (Supplementary Fig. 2-4). For completeness, mean E2A is reported (Supplementary Fig. 4), with mean E2A = 1.7° ± 7.1°.

**Fig. 4:**
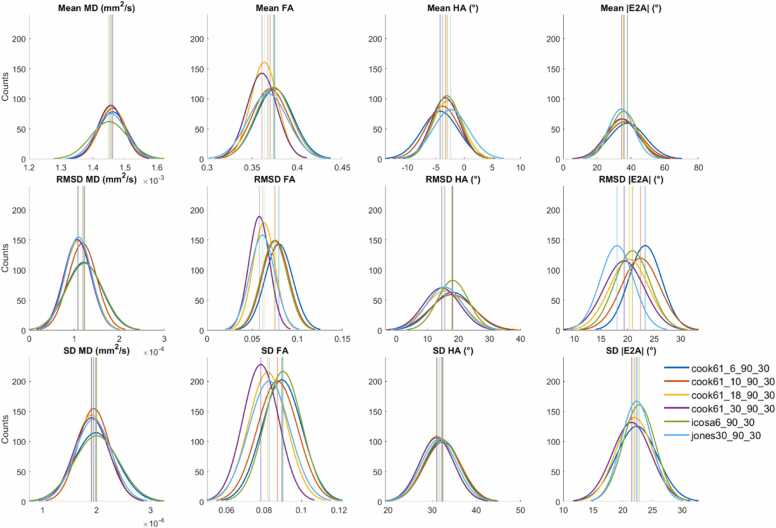
Histograms of (top to bottom) mean, RMSD, and SD of (left to right) MD, FA, HA, and |E2A| across 500 bootstrap samples and healthy volunteers (N = 10). Data from six time-normalized acquisition schemes are presented, with vertical lines indicating 95% confidence intervals of the mean. Non-overlapping 95% CI indicate significant differences between groups. *MD* mean diffusivity, *FA* fractional anisotropy, *HA* helix angle, *|E2A|* absolute sheetlet angle, *RMSD* root mean squared difference, *SD* standard deviation, *CI* confidence intervals

**Fig. 5:**
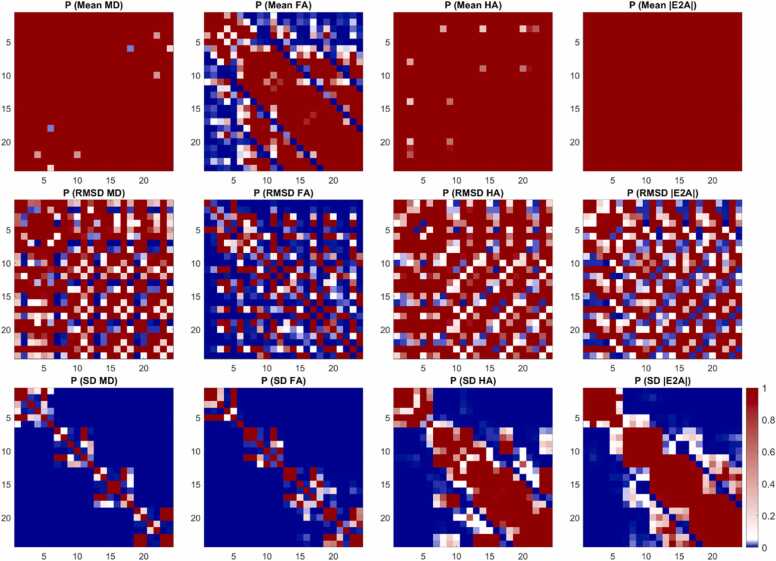
P-value matrices reflecting pairwise comparisons between the 24 acquisition schemes given in Fig. 3 with p < 0.05 (blue), p = 0.05 (white), and p > 0.05 (red). *MD* mean diffusivity, *FA* fractional anisotropy, *HA* helix angle, *|E2A|* absolute sheetlet angle, *RMSD* root mean squared difference, *SD* standard deviation

A subset of 6 acquisition schemes corresponding to NA_all_ = NA_b500_ + NA_b50_ = 120 are presented as histograms (Fig. 4). RMSD and SD for MD, FA, HA, and |E2A| were significantly lower for ND ≥ 18 compared to ND = 6 data, indicating better accuracy and precision in DES with greater number of directions rather than repetitions. This applied across Cook, icosahedral, and Jones diffusion schemes. P-value matrices illustrate significant differences (p < 0.05) between acquisition schemes, which were most prominent in precision (SD) and least prominent in mean values across different ND and NA (Fig. 5).

**Fig. 6:**
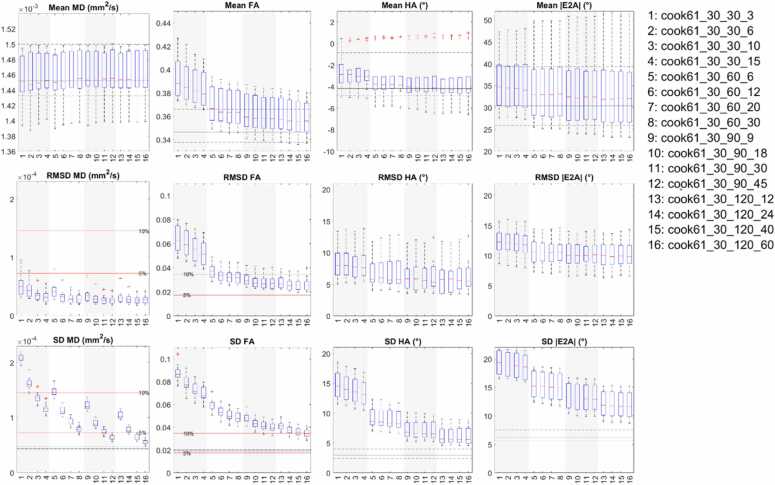
Boxplots of cDTI metrics (left to right) MD, FA, HA, and |E2A| showing (top) cDTI metrics averaged over bootstrap samples, (middle) RMSD with respect to the fully sampled reference data, and (bottom) SD across bootstrap samples across a mid-myocardial short-axis slice. Sixteen acquisition schemes were sorted by number of low b-value acquisitions (NA_b50_) and grouped into four groups (white and gray vertical bands) with increasing NA_all_ corresponding to increasing acquisition times. For reference, median and IQR values from the reference dataset are given (black solid and dashed lines); 5% and 10% of the median MD and FA from the reference dataset are indicated (red solid lines). *cDTI* cardiac diffusion tensor imaging, *MD* mean diffusivity, *FA* fractional anisotropy, *HA* helix angle, *|E2A|* absolute sheetlet angle, *RMSD* root mean squared difference, *SD* standard deviation, *NA* number of acquisitions

We also report the mean, RMSD, and SD for the four cDTI parameters as a function of numbers of low and high b-value acquisitions, NA_b50_, and NA_b500_. For clarity, only a single DES Cook61_30 is presented (Fig. 6). For context, these schemes are highlighted in bold in Table 1. Within each band of fixed NA_b500_, there was a trend toward lower RMSD and SD of MD and FA. A similar trend was observed in RMSD and SD of HA and |E2A| at lower total number of acquisitions (NA_all_ < 45), but was not discernible at higher NA_all_. For the same NA_b500_, the schemes with NA_b500_:NA_b50_ = 2 had on average, RMSD MD, and FA that were 29% and 13% lower than the NA_b500_:NA_b50_ = 10 schemes. Similarly, SD MD and FA were 46% and 20% lower in the former compared to the latter.

**Fig. 7:**
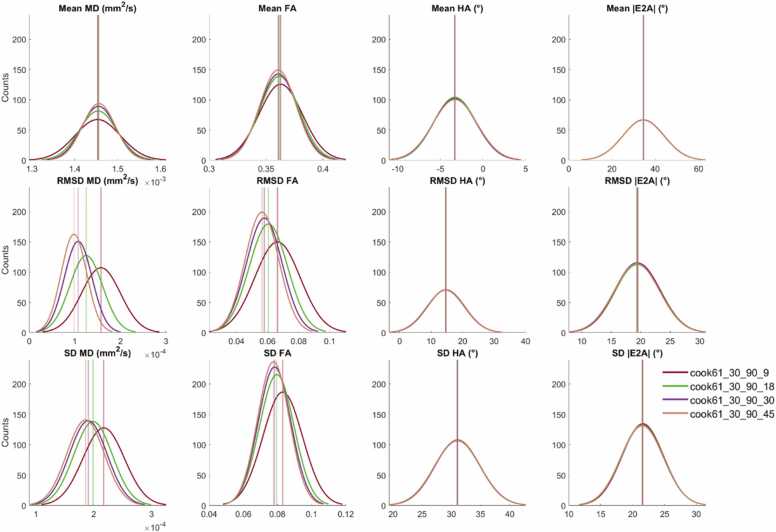
Histograms of (top to bottom) mean, RMSD and SD of (left to right) MD, FA, HA, and |E2A| across 500 bootstrap samples and healthy volunteers (N = 10). Data from a single diffusion encoding scheme with different numbers of low b-value acquisitions (NA_b50_ = 9, 18, 30, 45) are presented, with vertical lines indicating 95% confidence intervals of the mean. Non-overlapping 95% CI indicate significant differences between groups. *MD* mean diffusivity, *FA* fractional anisotropy, *HA* helix angle, *|E2A|* absolute sheetlet angle, *RMSD* root mean squared difference, *SD* standard deviation, *NA* number of acquisitions, *CI* confidence intervals

**Fig. 8:**
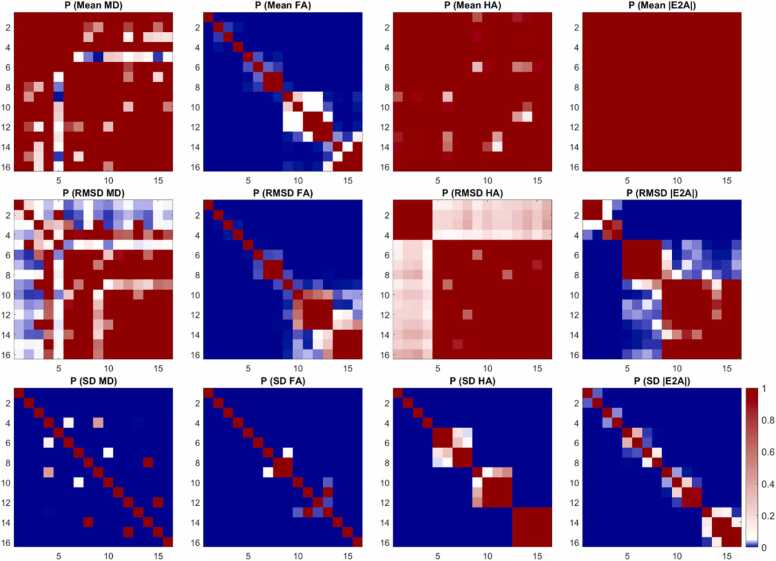
P-value matrices reflecting pairwise comparisons between the 16 acquisition schemes given in Fig. 6 with p < 0.05 (blue), p = 0.05 (white), and p > 0.05 (red). *MD* mean diffusivity, *FA* fractional anisotropy, *HA* helix angle, *|E2A|* absolute sheetlet angle, *RMSD* root mean squared difference, *SD* standard deviation

A subset of a single DES with four sets of NA_b50_ are presented as histograms (Fig. 7). RMSD and SD of MD and FA were significantly lower for NA_b50_ ≥ 18 compared to NA_b50_ ≤ 10 data, indicating better accuracy and precision with greater number of low b-value acquisitions. No significant differences were observed in RMSD and SD of HA and |E2A|. P-value matrices illustrate significant differences (p < 0.05) that were most prominent in accuracy (RMSD) and precision (SD) of MD and FA across different NA_b50_ and NA_all_ (Fig. 8).

Data in the mid-ventricular septal wall are presented (Supplementary Figs. 5 and 6). Notwithstanding the larger error bars due to fewer voxels, similar trends were observed as compared to the whole mid-ventricular slice data. This suggests that structured noise in regions more greatly affected by susceptibility effects, i.e., near the posterior vein, did not detract from the overall findings.

**Fig. 9:**
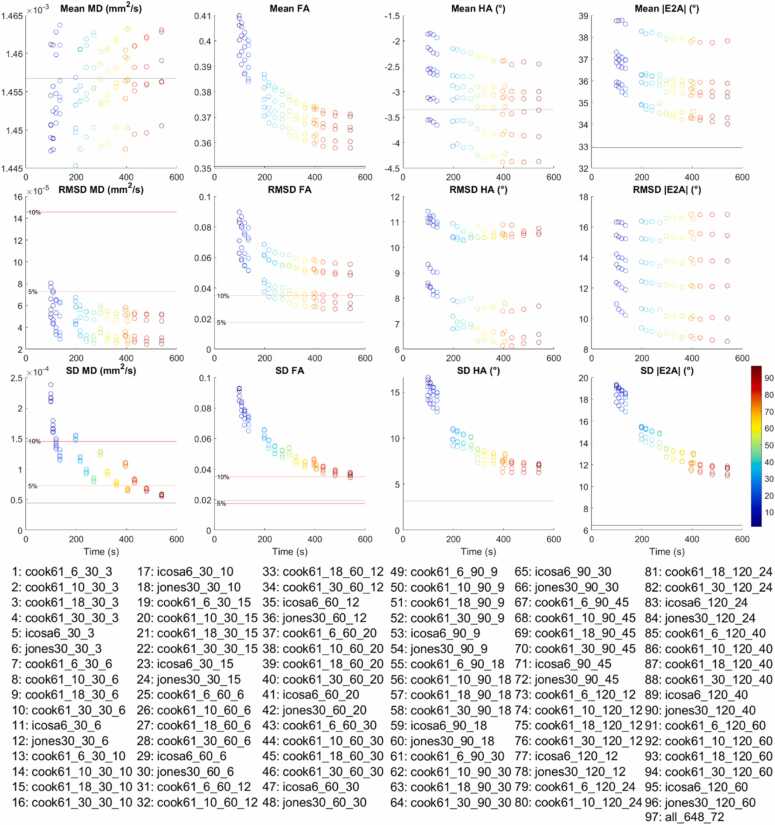
Mean, RMSD, and SD of MD, FA, HA, and |E2A| within a mid-myocardial slice averaged across subjects and plotted against nominal acquisition time. Each acquisition scheme 1–97 is encoded by color and described in the legend. For better presentation, mean values for the reference dataset labeled “all_648_72” are not plotted due to long scan time, and are instead given by black lines; 5% and 10% of the mean MD and FA from the reference dataset are indicated by red lines. *MD* mean diffusivity, *FA* fractional anisotropy, *HA* helix angle, *|E2A|* absolute sheetlet angle, *RMSD* root mean squared difference, *SD* standard deviation

Average mean, RMSD, and SD of cDTI parameters were evaluated as a function of acquisition time (Fig. 9). The data show decreasing RMSD and SD with increasing NA_all_ and scan time. The majority of DES investigated yielded an RMSD MD of <5% and SD MD of <10% of the reference MD. Acquisition schemes DES_All__90_45, DES_All__120_40, and DES_All__120_60 yielded SD MD of <5% of the reference MD, where DES_All_ corresponded to all DES. Differences in MD between time-normalized DES were marginal. DES were ranked from most to least accurate FA (i.e., lowest to highest RMSD): Cook61_30, Jones30, Cook61_18, Icosa6, Cook61_10, Cook61_6, with RMSD FA <10% of the reference FA for Cook61_30/Jones30 with NA_all_ ≥ 108. SD FA <10% of the reference FA for Cook61_30_120_60 only. DES were ranked (i) from most to least accurate HA: Cook61_30, Jones30, Cook61_18, Cook61_10, Cook61_6, Icosa6, (ii) from most to least precise HA: Cook61_30, Cook61_18, Jones30, Icosa6, Cook61_10, Cook61_6, (iii) from most to least accurate |E2A|: Jones30, Cook61_30, Cook61_18, Icosa6, Cook61_10, Cook61_6, and (iv) from most to least precise |E2A|: Jones30, Icosa6, Cook61_10, Cook61_6, Cook61_18, Cook61_30. NA_b500_ = 120 and Cook61_30/Cook61_18 was needed to achieve SD HA < 7°, while SD |E2A| < 13° was achievable with NA_b500_ = 120 and Jones30, Icosa6.

Higher NA_all_, i.e., longer acquisition times led to improved precision in all DTI metrics whereby quadrupling NA_all_ from 40 to 160 volumes led to *a* 53%, 50%, 53%, and 36% reduction in SD in MD, FA, HA, and |E2A|, averaged across 6 DES. In relative terms, this corresponded to *a* reduction in SD MD from 9.7% to 4.6% and SD FA from 22% to 11%, expressed as *a* percentage of the mean MD and FA of the reference data. Similarly, accuracy improved with *a* 20%, 39%, 11%, and 5.4% reduction in RMSD in MD, FA, HA, and |E2A| from 40 to 160 acquired volumes. In relative terms, this corresponded to a reduction in RMSD MD from 3.3% to 2.7% and RMSD FA from 19% to 12% of the reference mean MD and FA. RMSD and SD in selected acquisition schemes are given in Table 2.

**Table 2:** RMSD and SD of MD (×10^−4^ mm^2^/s), FA, HA (°), |E2A| (°) for selected acquisition schemes, varying (top to bottom) NA_b50_, NA_all_, and diffusion encoding scheme (DES; mean ± SD over 10 subjects).

Vary NA_b50_		76: Cook 61_30_120_12	82: Cook 61_30_120_24	88: Cook 61_30_120_40	94: Cook 61_30_120_60		97: Reference
RMSD	MD	0.33±0.12	0.29±0.09	0.27±0.07	0.28±0.08		0
	FA	0.028±0.006	0.027±0.006	0.026±0.006	0.027±0.006		0
	HA	6.1±2.7	6.1±2.7	6.2±2.7	6.3±2.8		0
	|E2A|	10±2	10±2	10±2	10±2		0
SD	MD	1.04±0.07	0.77±0.06	0.64±0.05	0.55±0.05		0.448±0.037
	FA	0.042±0.004	0.038±0.004	0.036±0.004	0.035±0.004		0.020±0.002
	HA	6.3±1.5	6.3±1.5	6.3±1.5	6.3±1.5		3.2±0.9
	|E2A|	12±2	12±2	12±2	12±2		6±1

Vary NA_all_		16: Cook 61_30_30_10	40: Cook 61_30_60_20	64: Cook 61_30_90_30	88: Cook 61_30_120_40		
RMSD	MD	0.36±0.11	0.30±0.09	0.28±0.08	0.27±0.07		
	FA	0.055±0.011	0.033±0.007	0.028±0.006	0.026±0.006		
	HA	8.1±2.5	6.9±2.7	6.3±2.5	6.2±2.7		
	|E2A|	12±2	11±2	10±2	10±2		
SD	MD	1.36±0.10	0.92±0.07	0.74±0.06	0.64±0.05		
	FA	0.073±0.007	0.050±0.006	0.041±0.005	0.036±0.004		
	HA	14±2	8.9±1.8	7.2±1.6	6.3±1.5		
	|E2A|	19±2	15±2	13±2	12±2		

Vary DES		85: Cook 61_6_120_40	86: Cook 61_10_120_40	87: Cook 61_18_120_40	88: Cook 61_30_120_40	89: Icosa 6_120_40	90: Jones 30_120_40
RMSD	MD	0.52±0.06	0.47±0.12	0.30±0.08	0.27±0.07	0.52±0.17	0.25±0.06
	FA	0.056±0.011	0.050±0.010	0.035±0.008	0.026±0.006	0.049±0.009	0.031±0.009
	HA	11±4	10±4	7.6±3.3	6.2±2.7	10.6±2.2	6.5±2.1
	|E2A|	17±2	16±3	12±2	10±2	13±2	9±2
SD	MD	0.69±0.06	0.68±0.06	0.65±0.05	0.64±0.05	0.68±0.05	0.68±0.05
	FA	0.039±0.004	0.040±0.005	0.037±0.005	0.036±0.004	0.039±0.004	0.038±0.004
	HA	7.3±1.9	7.3±2.2	6.6±1.9	6.3±1.5	7.1±1.2	7.1±1.5
	|E2A|	12±2	12±2	12±2	12±2	11±2	11±2

For ease of reference, the acquisition schemes were numbered according to the legend in Fig. 9

*MD* mean diffusivity, *FA* fractional anisotropy, *HA* helix angle, *|E2A|* absolute sheetlet angle, *RMSD* root mean squared difference, *SD* standard deviation

## 4 Discussion

We evaluated 96 acquisition schemes in terms of accuracy and precision with respect to the 720 volumes, i.e., ∼36-minute reference datasets. First, we observed that accuracy and precision in all estimates improved with NA_all_ and acquisition time, consistent with previous reports [23], [35]. Second, we found that prioritizing ND over NR given a fixed acquisition time improved accuracy and precision in general. Differences in accuracy and precision between different DES with equal ND and NA_all_ were less prominent and generally not significant.

In our pilot work in healthy volunteers [35], we reported RMSD MD = 2.29 (×10^−4^ mm^2^/s), RMSD FA = 0.08, and RMSD HA = 13° using a Jones scheme with ND = 6, NA_b50_ = 24, and NA_b500_ = 96. The accuracy improved to RMSD MD = 1.11 (×10^−4^ mm^2^/s), RMSD FA = 0.04, and RMSD HA = 7.8° when using a Caruyer scheme [51] with a larger number of directions, i.e., ND = 96 and NR = 1. It was concluded that precision in cDTI was improved by prioritizing ND over NR for a given acquisition time. This was supported by more recent work evaluating 6 ≤ ND ≤ 30, where it was reported that increasing ND led to reduced RMSD in cDTI parameters, and that recommended an experimental design strategy that maximizes ND [36]. In the current study, the nearest equivalent acquisition scheme to the former was Cook61_6_90_30 which yielded RMSD MD = 0.794 × 10^−4^ mm^2^/s, RMSD FA = 0.04, and RMSD HA = 8.4°. This represented an improvement in accuracy compared to the previous study, which may be attributed to the improved post-processing pipeline with robust fitting of tensors [52] over shot rejection. Direct comparison to a 96-direction DES was not available. Another pilot study in healthy volunteers (N = 5) explored time-normalized cDTI acquisitions with six DES where ND = 6, 10, 12, 15, 20, 30 and NR = 10, 6, 5, 4, 3, 2, respectively [34]. The authors reported minimal differences in median and interquartile intervals of MD and FA, and they suggested that the acquisition scheme was not critical to measuring MD and FA. In a separate study using stimulated echo acquisition mode (STEAM), no significant differences were found in MD and FA between time-normalized averaged datasets where ND = 6, 10, 12, and 20 [53]. Our results suggest otherwise and indicate that prioritizing ND over NR for a given scan time, up to ND = 30, improves accuracy and precision of cDTI parameters. For example, RMSD and SD in MD and FA were significantly lower in Cook61_30_120_40 compared to Cook61_6_120_40 (*p* < 0.001). Moreover, there is potential for poor image quality associated with specific diffusion encoding directions due to eddy current effects. Acquiring the minimum of ND = 6 provides no redundancy in case a specific diffusion encoding direction gives rise to sub-optimal image quality.

The effects of ND have also been reported ex vivo [23], [54]. One study in fixed pig hearts found that reliable estimation of HA could be obtained with ND/NR = 12/6, 30/3, or 64/2, recommending the 12/6 combination as one with the shortest (<10 min) acquisition time [54]. However, only qualitative assessments were reported and data were not time-normalized. A second study in fixed rat hearts investigated the effects of ND, SNR, and spatial resolution on the accuracy and precision of cDTI [23]. It was shown that for a given scan time, the precision of FA, HA, |E2A|, transverse angle, sheetlet elevation, and sheetlet azimuth for *a* given scan time were largely independent of the choice of increasing NR or ND, on the assumption that SNR is proportional to √NR. In practice, physiological effects in vivo, such as residual motion effects arising from breathing and cardiac contraction, contribute *a* significant additional noise-like component that violates the above assumption, resulting in spatially correlated variations in parameters. This may, to an extent, reduce the value of additional NR and support our current findings of prioritizing ND over NR, up to ND = 30. Extrapolating to typical parameter settings, e.g., resolution used in the clinic, the study suggested that the expected bias in MD and FA were 2.1% and 13% and precision in MD and FA were ±4.9% and ±30% with respect to the ground truth; precision of HA and |E2A| were ±14°and ±24°, respectively. This compares well with our current findings of bias in MD and FA of 2.7% and 12% and precision in MD and FA of 4.6% and 11% for an NA = 160 dataset with respect to the reference data, noting that the previous study reported precision over voxels rather than bootstrap samples. Direct comparisons between ex vivo and in vivo data are difficult due to various reasons, such as different physiological status and temperature of the myocardium. Nonetheless, our current findings similarly reflect that precision of MD is superior to that of FA, and precision of HA is superior to that of |E2A|, although the specific values differ. While *a* ground truth does not exist in vivo, we can see that the trends in accuracy and precision appear to approach an asymptotic value by NA_all_ = 160, justifying the appropriateness of NA_all_ = 720 as suitable reference data.

In the above rat heart study, precision in MD was optimized by maximizing NR of non-diffusion-weighted scans at the expense of ND, for *a* given scan time. This leads us to our third observation that accuracy and precision of MD, and to *a* lesser extent FA, improved with increasing numbers of low *b*-value acquisitions NA_b50_, with disproportionately large benefits at low NA_all_. With larger NA_all_ ≥ 99, as more commonly used in the clinic, improvements in both accuracy and precision in MD and FA were retained. For example, with DES Cook61_30, NA_b500_ = 90 and NA_b50_ = [9, 18, 30, 45], the improvements in precision of MD and FA between each increasing step of NA_b50_ were significant (p < 0.001). Accuracy in MD improved when NA_b50_ increased from 9 to 18 (p < 0.05), but was not significantly better between successive increments of higher NA_b50_.

Other studies investigated the sensitivity of cDTI to SNR in human hearts [49] and in fixed rat hearts [55]. In the former, systolic median 95% CI of the first, second, and third eigenvectors (**v**_**1**_, **v**_**2**_, and **v**_**3**_) were 15.5°, 31.2°, and 21.8°. The authors concluded that precision improved with increasing SNR, but the improvements were minimal beyond NR = 10 corresponding to a 10-min scan. This was consistent with our findings. The latter study reported that mean 95%CI of **v**_**1**_, **v**_**2**_, and **v**_**3**_ was 3.7°, 10.9°, and 10.6°, respectively. The poorer precision in vivo may reflect the additional challenges of motion and lower spatial resolution in vivo. While higher image resolution in vivo is desirable to minimize partial-volume effects, this is generally limited by SNR, and we employed a moderate image resolution of 2.3 × 2.3 × 8 mm^3^ to maximize generalizability.

Precision and bias of cDTI measurements could potentially be affected by several factors. These include orientation and anisotropy of the underlying diffusion tensor [27], physiological “noise” due to residual cardiac and respiratory motion effects, and signal rectification due to insufficient SNR. Signal rectification can be mitigated by using weighted squares fitting, as well as complex image data reconstruction [22], [56], [57], [58]. However, these do not resolve the contribution of physiological “noise” which can be substantial.

The recommended protocols for cDTI will depend on several factors, including the precision of the measurement, the expected differences, e.g., between health and pathology, the parameters of interest, subject compliance, and scan time available. Despite our finding that higher NA_all_ improved precision, this improvement comes with diminishing returns at higher NA_all_. Moreover, longer scans, e.g., >10 min, can be challenging to perform in a clinical setting due to limited scan times and increased likelihood of patient discomfort leading to greater patient motion, poorer image quality, and premature termination of scans. For context, we consider the range of cDTI parameters seen in clinical cohorts. Where the differences between health and disease are known and expected to be small, the acquisition would need to be designed with greater precision. In general, studies report higher MD and lower FA in disease cohorts relative to controls. In a study of patients post-myocardial infarction [2], MD was 14% higher, FA was 31% lower, and HA was between −7° and +5° relative to controls. In patients with hypertrophic cardiomyopathy [7], [8], [10], [11], MD was between 2% and 10% higher, FA was between 6% and 17% lower relative to controls. Patients with amyloidosis had 26% higher MD and 29% lower FA relative to controls [13]. Similarly, patients with aortic stenosis had 6% higher MD and 17% lower FA relative to controls [12]. HA and E2A differences in pathology have been primarily reported in terms of HA slope or |E2A| [2], [7], [8], [10], [12], [59] and differences in HA may not be directly comparable. Other studies that reported |E2A| were based on STEAM [3], [9], which is known to yield substantially different measurements compared to spin echo. We additionally reported E2A instead of |E2A| to avoid loss of signed information that reflects on sheetlet orientation (Supplementary Fig. 4). This is particularly useful in assessing bias across different regions with positive and negative E2A. Averaging across the myocardium means that regions with positive and negative E2A cancel out resulting in mean E2A < mean |E2A|.

To achieve SD MD of <5% of the reference mean, NA_b50_ ≥ 45 with any DES was required. This included DES_All__90_45, DES_All__120_40, and DES_All__120_60 where DES_All_ refers to any of six DES evaluated. DES_All__90_45 had the shortest acquisition time of 6:45 min. In contrast, only Cook61_30_120_60 yielded SD FA of <10% of the reference, i.e., 9.8%, corresponding to an acquisition time of 9:00 min. Other sequences, e.g., Cook61_30_120_40, came close with SD FA = 10.2% with an acquisition time of 8:00 min. That SD FA > SD MD was consistent with the greater sensitivity of FA to noise. SD HA and SD |E2A| were more sensitive to NA_all_ and DES, and less so to NA_b50_. Besides extending the acquisition time, precision in HA and |E2A| could be improved by prioritizing ND over NR, e.g., ND = 30, gave higher precision than ND = 6, given the same total acquisition time. For clinically relevant protocols, our findings support the use of ND = 30. We would also recommend NA_b50_:NA_b500_ ≥ 1/3 for better precision in MD and FA. If only MD were desired, *a* shorter protocol of <7 min would be reasonable. We would recommend protocols of >8 min to obtain good precision for FA, HA, and |E2A|, subject to availability of scan time.

## 5 Limitations

In this study, we have only considered healthy volunteers with good compliance over the entire ∼36 min scan, excluding setup and planning. It is foreseeable that some patients will have poorer compliance leading to poorer image quality, accuracy, and precision, suggesting more acquisition volumes and time would be needed to achieve similar levels of accuracy and precision. However, longer acquisition times are likely to contribute to worse compliance and may be infeasible. In the ideal case, we would examine accuracy and precision in a similar study in patients. In practice, such efforts may need to be combined with techniques for accelerating image acquisition, such as simultaneous multi-slice imaging [60], compressed sensing [61], [62], and deep learning [63], [64], [65], [66].

Image distortion remains a common issue in cDTI. This stems mostly from the use of single-shot EPI readouts and consequent sensitivity to susceptibility-induced distortion. This commonly manifests as artifactual compression or dilation of the myocardium, especially near the posterior vein. In this study, we have seen that this effect also contributes to reduced local accuracy and precision. Recent developments in distortion correction using reversed phase encoding data for correction of susceptibility artifacts promise to improve the geometric fidelity of the images [67]. Distortions can also be caused by eddy current effects and can be an issue particularly in M2SE cDTI where large diffusion encoding gradients are used [22]. Methods based on gradient impulse response functions and diffusion gradient response functions [68] and potentially gradient system transfer functions [69] can be an effective tool in correcting for eddy current effects.

In this study, we used a free-breathing approach without respiratory navigators and/or slice tracking. This suggests that the data would have been susceptible to through-slice motion, which we estimate to within ±1.5 mm. This was mitigated through the use of robust fitting to exclude outliers [41], the use of thick 8 mm slices, and acquisition of multiple diffusion encoding directions and repetitions, yielding high-quality DTI maps. While some studies have adopted approaches for managing through-slice motion, e.g., breath-holding [22], respiratory navigators [14], [70], and/or slice tracking [19], these techniques either reduce scan efficiency and/or rely on assumptions of heart geometry and motion, and are an incomplete solution. Compensation for respiratory motion in cDTI remains an area of ongoing development. Furthermore, M2SE cDTI is sensitive to cardiac phase [49], and this study focused on imaging in late systole when the acquisition is most robust.

To maximize the number and scope of acquisition schemes compared, we relied partially on subsampling of the modified Cook61 DES. This meant that (i) the subsampled data were not fully independent of each other, and (ii) the distribution of diffusion encoding directions in subsampled schemes was not fully optimal. To mitigate these, fully optimized Icoas6 and Jones30 schemes were employed as independent controls. Their accuracy and precision were seen to be comparable to the Cook61_6 and Cook61_30 schemes, respectively. Despite our approach, which enabled systematic comparison of a relatively large number of acquisition schemes, we were limited to ND = 30 due to scan time constraints. Some advanced applications, e.g., resolving crossing fibers in the brain, require larger ND. However, our findings suggest diminishing returns in cDTI with increasing ND > 30, and there is limited evidence for higher ND, particularly where voxels with discrete crossing cell populations are limited in the myocardium. An alternative would be to employ acquisition schemes that are completely independent, as in previous pilot studies [53], [54]. However, this approach extends the imaging time, limits the number of acquisition schemes that can be evaluated, and often means that datasets cannot be time-normalized. Even so, the common approach of combining all data to form a reference dataset means that reference data are not completely independent of the component data.

## 6 Conclusions

In summary, we investigated the trade-off between the number of diffusion encoding directions and repetitions in M2 spin echo-based cDTI. For a given acquisition time, we found that prioritizing the number of diffusion encoding directions over NR generally yields better accuracy and precision in cDTI parameters, particularly MD and FA. We also observed that greater sampling of low b-value data improves accuracy and precision of MD and FA but not HA and |E2A|. Finally, we characterized the improvements in accuracy and precision associated with increasing the total number of acquisitions. These results may serve to guide optimization of protocols for supporting ongoing efforts in harmonization and standardization of cDTI and aid its development toward wider clinical adoption.

## Funding

This work was supported by the 10.13039/501100000274British Heart Foundation, UK (PG/19/1/34076, CH/16/2/32089), and the 10.13039/100004440Wellcome Trust (219536/Z/19/Z).

## Author contributions

**Sven Plein:** Writing – review & editing, Supervision, Project administration, Funding acquisition. **Jürgen E. Schneider:** Writing – review & editing, Resources, Funding acquisition. **Erica Dall’Armellina:** Writing – review & editing, Supervision, Project administration, Funding acquisition. **Irvin Teh:** Writing – review & editing, Writing – original draft, Supervision, Resources, Project administration, Methodology, Investigation, Funding acquisition, Formal analysis, Data curation, Conceptualization. **Christopher Nguyen:** Writing – review & editing, Software. **David Shelley:** Writing – review & editing, Project administration, Data curation. **Sam Coveney:** Writing – review & editing, Writing – original draft, Software, Methodology, Formal analysis. **Maryam Afzali:** Writing – review & editing. **Richard J. Foster:** Writing – review & editing, Data curation. **Noor Sharrack:** Writing – review & editing, Supervision, Project administration. **Ana-Maria Poenar:** Writing – review & editing, Supervision, Project administration.

## Ethics approval and consent

The study was conducted in accordance with the Declaration of Helsinki and was approved by the UK National Research Ethics Service (19/YH/0324; 18/YH/0168). All subjects provided written informed consent.

## Consent for publication

Not applicable.

## Declaration of competing interests

The authors declare that they have no known competing financial interests or personal relationships that could have appeared to influence the work reported in this paper.

## Data Availability

The datasets used and/or analyzed during the current study are available from the corresponding author on reasonable request.
