# Supplementary material for: Optimizing cardiac diffusion tensor imaging in vivo: More directions or repetitions?
Source: J Cardiovasc Magn Reson. 2025 Sep 2;27(2):101951. doi: 10.1016/j.jocmr.2025.101951 (PMC12702110; doi:10.1016/j.jocmr.2025.101951)
Supplement: Supplementary file 2 — Supplementary material [file mmc1.docx]

**SUPPLEMENTARY INFORMATION**


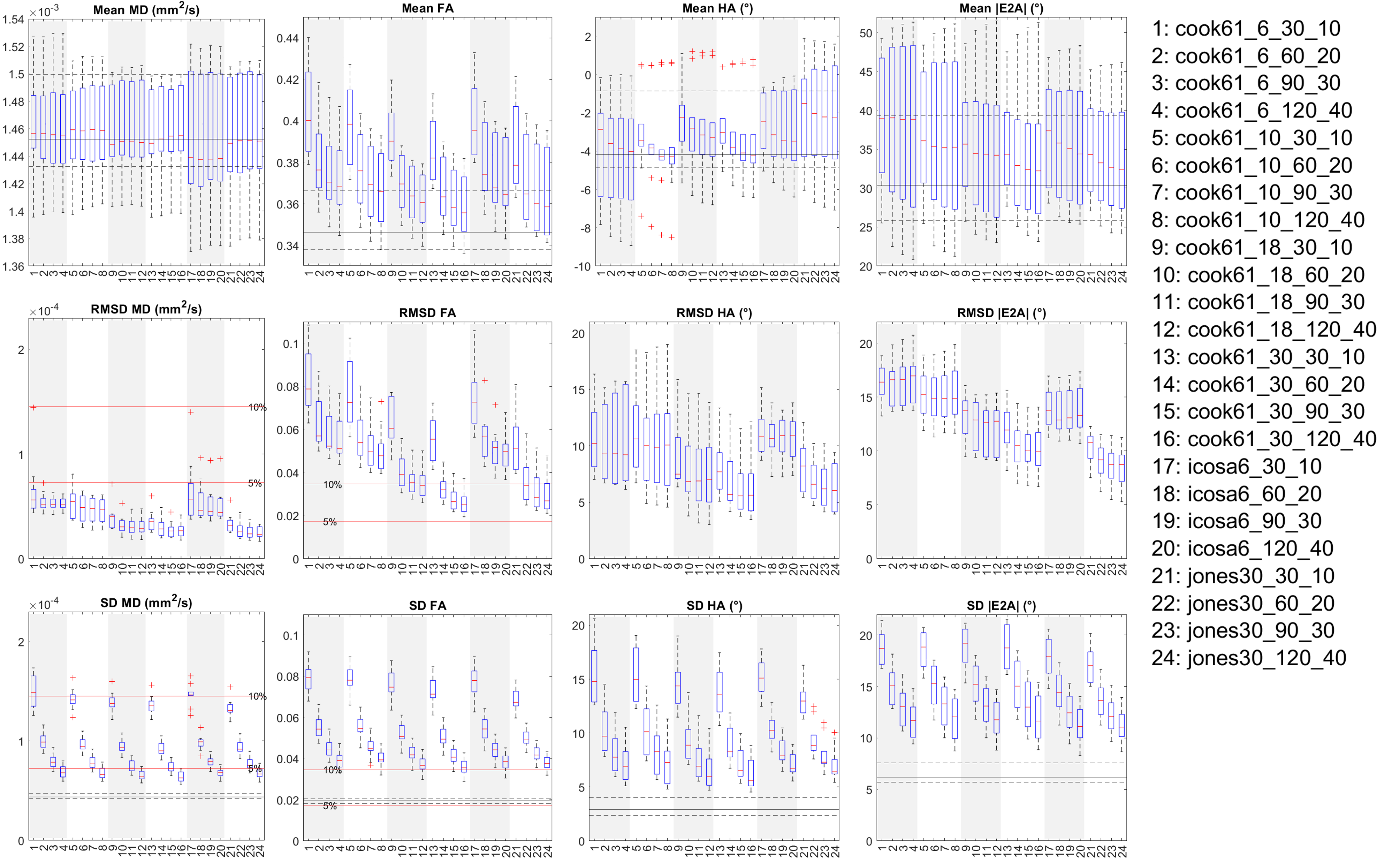


Supplementary Figure 1. Boxplots of cDTI metrics (left to right) MD, FA, HA and |E2A| showing (top) cDTI metrics averaged over bootstrap samples, (ii) RMSD with respect to the fully sampled reference data, and (iii) SD across bootstrap samples across a mid-myocardial short-axis view. 24 diffusion encoding schemes were sorted by total number of acquisitions (NA_all_) and grouped into six groups (white and grey vertical bands) with different diffusion encoding schemes. For reference, median and IQR values from the reference dataset are given (black solid and dashed lines); 5% and 10% of the median MD and FA from the reference dataset are indicated (red solid lines).


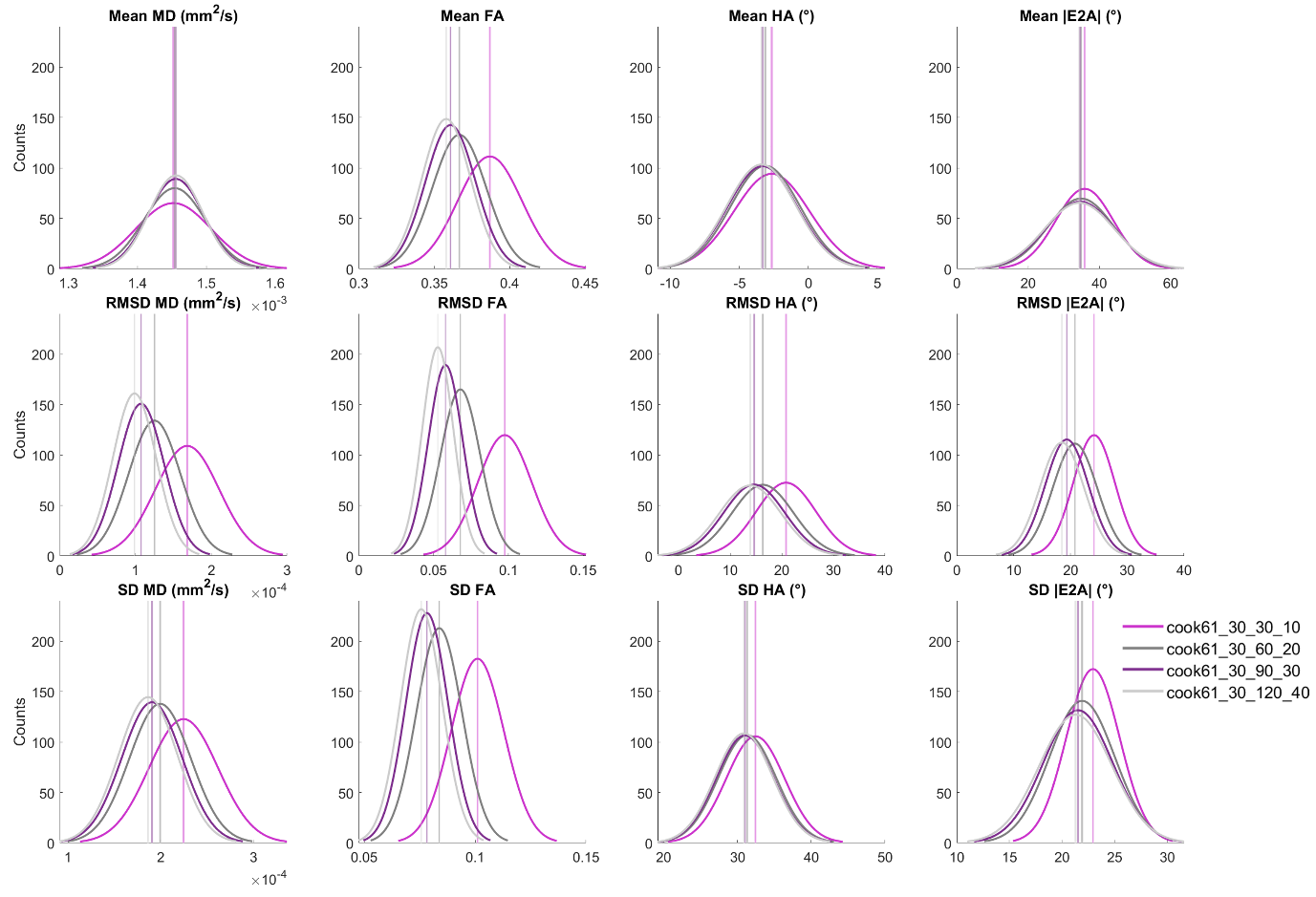


Supplementary Figure 2. Histograms of (top to bottom) mean, RMSD and SD of (left to right) MD, FA, HA and |E2A| across 500 bootstrap samples and healthy volunteers (N = 10). Data from a single diffusion encoding scheme with different numbers of low and high b-value acquisitions (NA_b500_ = 30, 60, 90, 120; NA_b50_ = 10, 20, 30, 40) are presented, with vertical lines indicating 95% confidence intervals of the mean. Non-overlapping 95% CI indicate significant differences between groups.


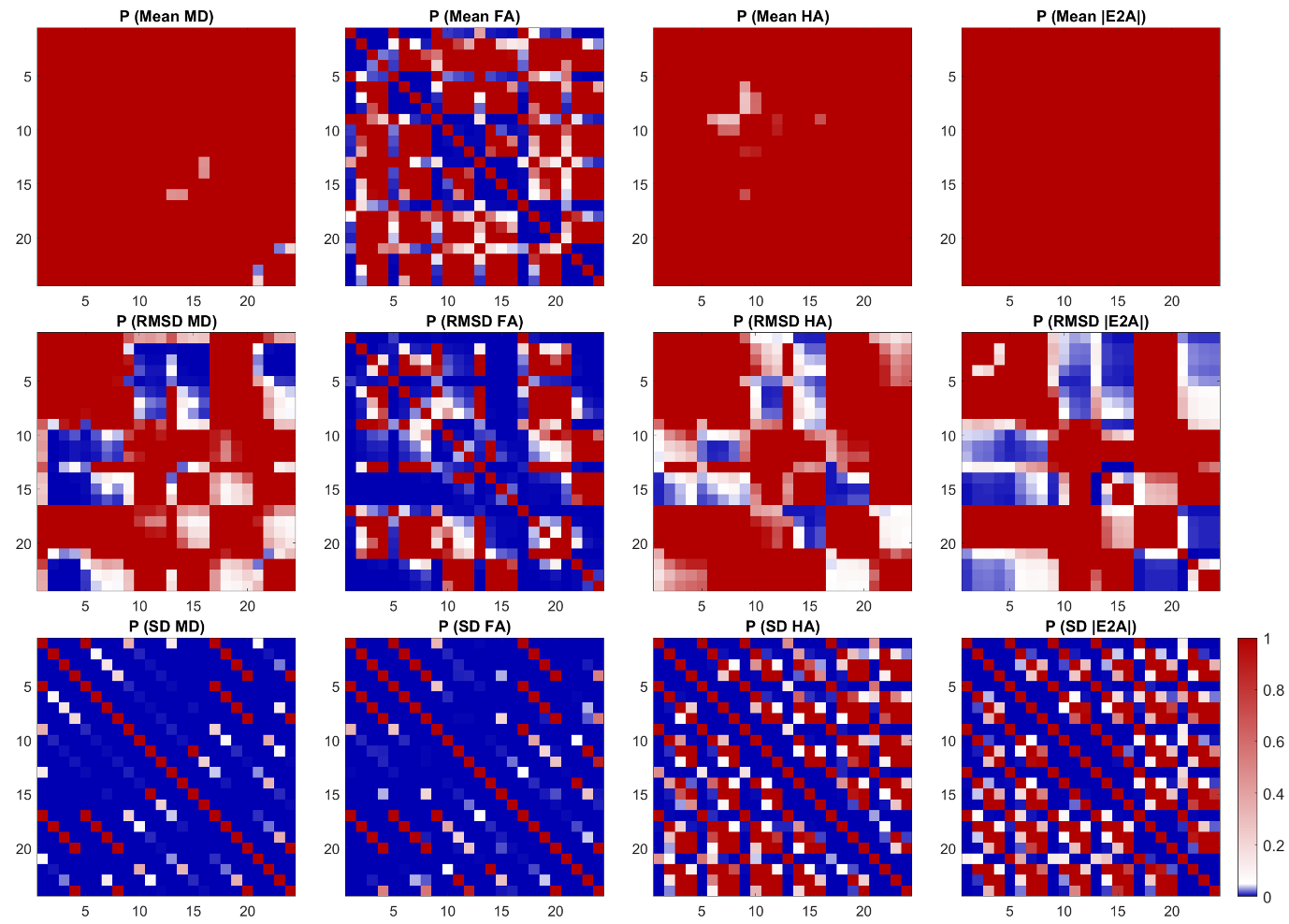


Supplementary Figure 3. P-value matrices reflecting pairwise comparisons between the 24 acquisition schemes given in Supplementary Figure 1 with p < 0.05 (blue), p = 0.05 (white) and p > 0.05 (red).


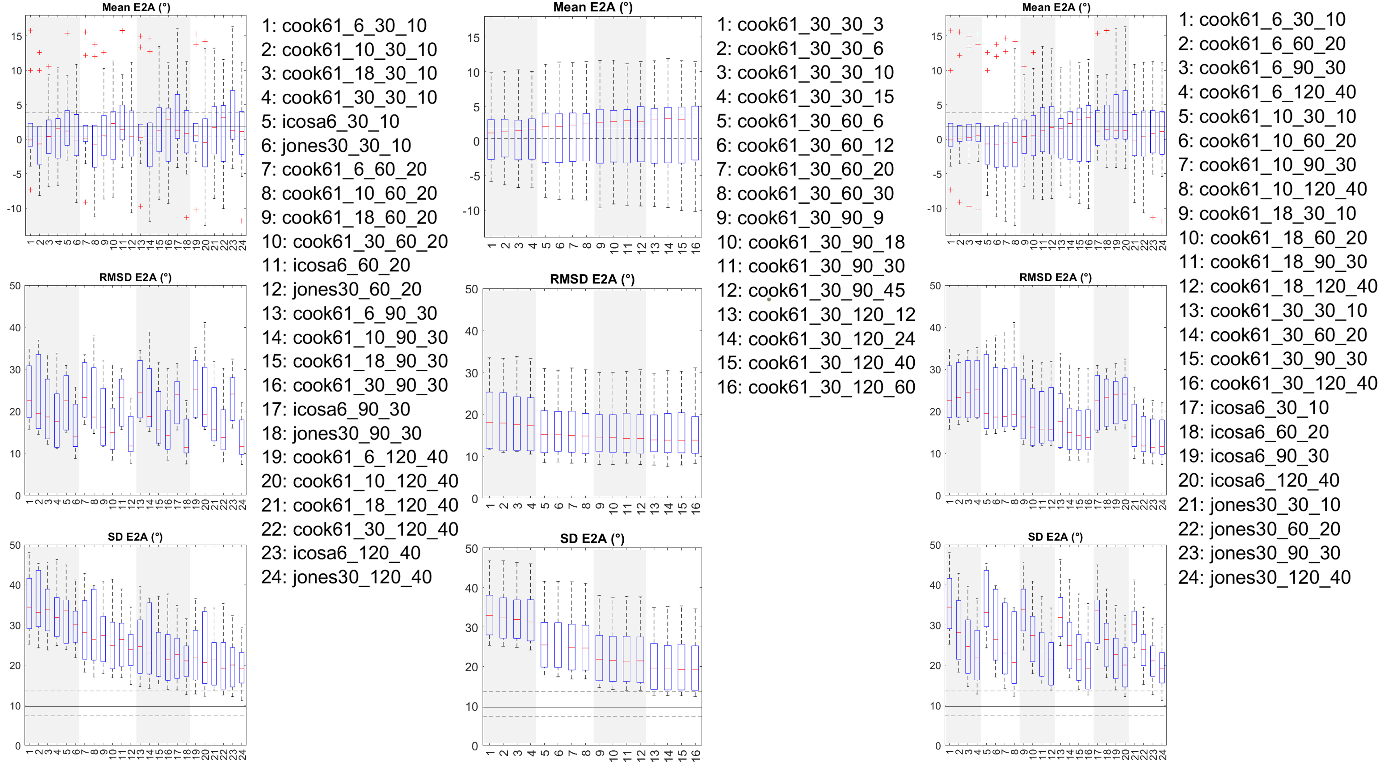


Supplementary Figure 4. Boxplots of mean E2A (top) averaged over bootstrap samples, (middle) RMSD with respect to the fully sampled reference data, and (bottom) SD across bootstrap samples across a mid-myocardial short-axis slice. (Left to right) Three sets of acquisition schemes as described by respective legends. For reference, median and IQR values from the reference dataset are given (black solid and dashed lines).


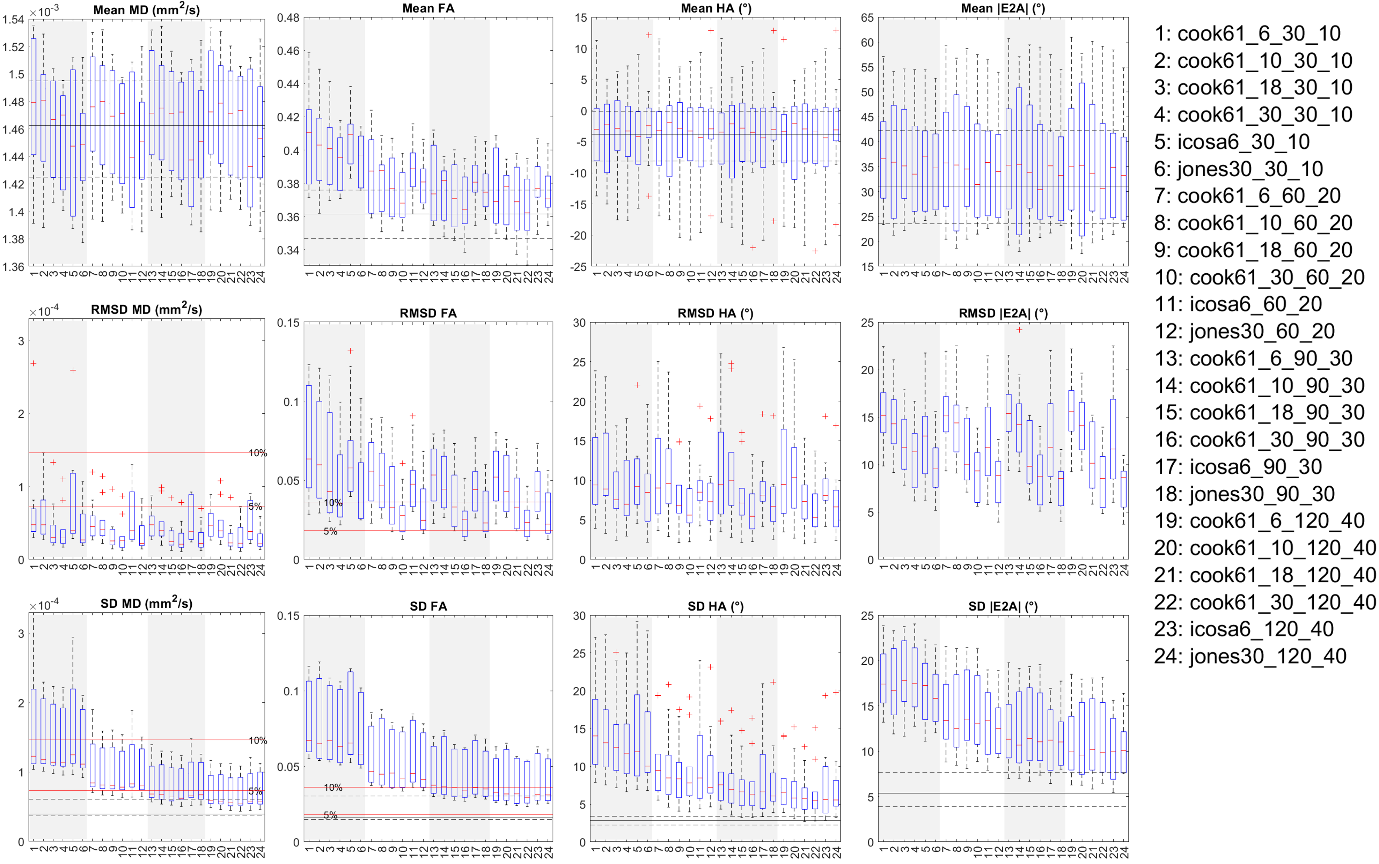


Supplementary Figure 5. Boxplots of cDTI metrics (left to right) MD, FA, HA and |E2A| in the *mid-ventricular septal wall (AHA regions 8 and 9)* showing (top) cDTI metrics averaged over bootstrap samples, (middle) RMSD with respect to the fully sampled reference data, and (bottom) SD across bootstrap samples. 24 acquisition schemes are described in the following format “DES_NA_b500__NA_b50_”, e.g. Cook61_30_90_30. These were sorted by diffusion encoding scheme and grouped into four groups (white and grey vertical bands) with increasing NA_all_ corresponding to increasing acquisition times. For reference, median and IQR values from the reference dataset are given (black solid and dashed lines); 5% and 10% of the median MD and FA from the reference dataset are indicated (red solid lines).


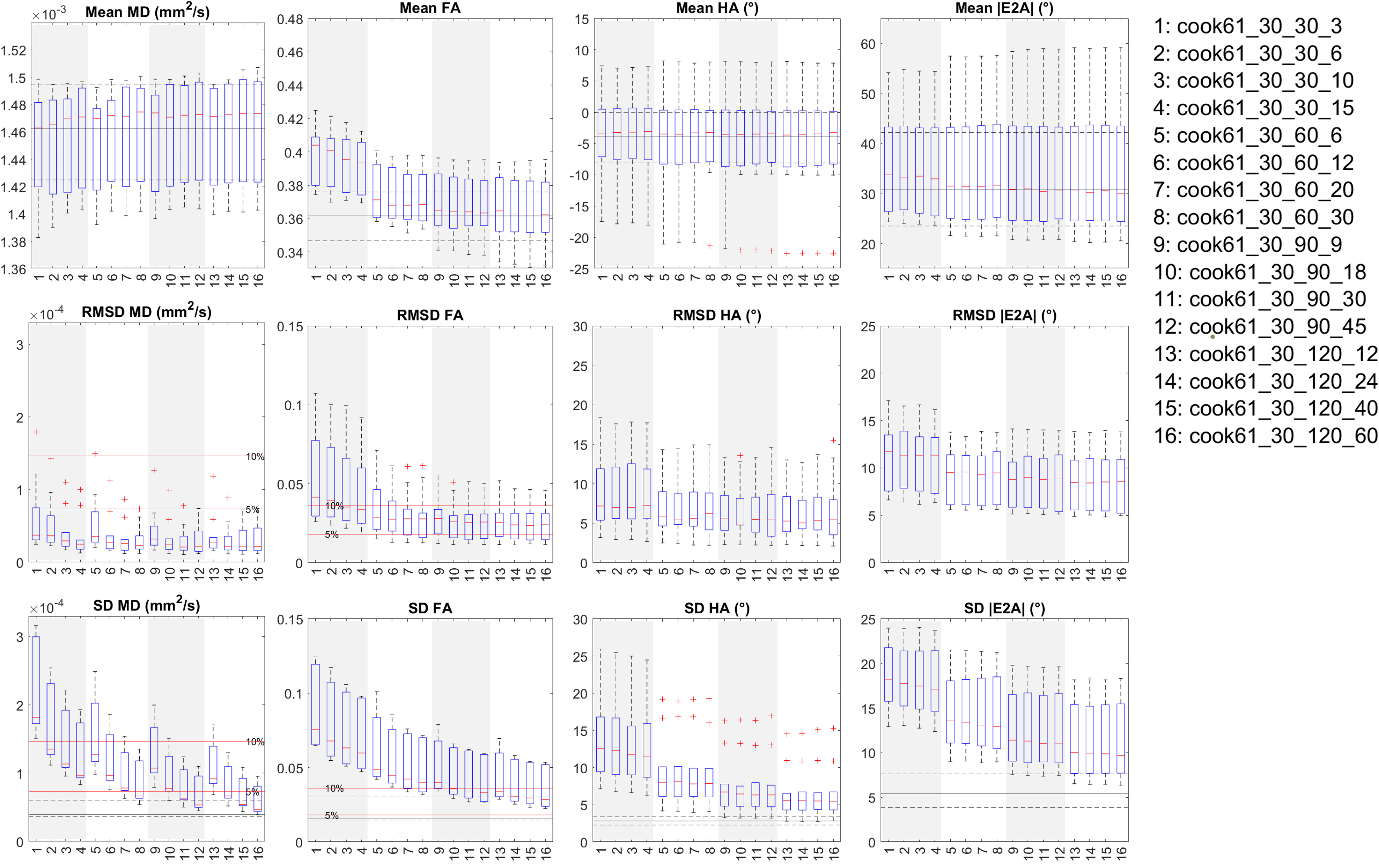


Supplementary Figure 6. Boxplots of cDTI metrics (left to right) MD, FA, HA and |E2A| in the *mid-ventricular septal wall (AHA regions 8 and 9)* showing (top) cDTI metrics averaged over bootstrap samples, (middle) RMSD with respect to the fully sampled reference data, and (bottom) SD across bootstrap samples. 16 acquisition schemes were sorted by number of low b-value acquisitions (NA_b50_) and grouped into four groups (white and grey vertical bands) with increasing NA_all_ corresponding to increasing acquisition times. For reference, median and IQR values from the reference dataset are given (black solid and dashed lines); 5% and 10% of the median MD and FA from the reference dataset are indicated (red solid lines).

Supplementary Video 1. Diffusion-weighted images (left and right) pre- and post-registration in a representative volunteer.
